# The Connection Between Resistance Training, Climbing Performance, and Injury Prevention

**DOI:** 10.1186/s40798-024-00677-w

**Published:** 2024-01-19

**Authors:** Atle Hole Saeterbakken, Nicolay Stien, Helene Pedersen, Kaja Langer, Suzanne Scott, Michail Lubomirov Michailov, Gudmund Gronhaug, Jiří Baláš, Tom Erik Jorung Solstad, Vidar Andersen

**Affiliations:** 1 Department of Sport, Food and Natural Sciences, Faculty of Education, Western Norway University of Applied Sciences, Campus Sogndal, Røyrgata 6, 6856 Sogndal, Norway; 2 Department of Human Sciences, Institute of Sports Science, Technical University Darmstadt, Darmstadt, Germany; 3 School of Anatomy, Faculty of Health and Life Sciences, University of Bristol, Bristol, UK; 4 Department Theory and Methodology of Sports Training, National Sports Academy, Sofia, Bulgaria; 5 Faculty of Physical Education and Sport, Charles University, Prague, Czech Republic

**Keywords:** Bouldering performance, Lead climbing, Maximal strength, Muscle hypertrophy, Muscular power, Local muscular endurance, Prevent injuries

## Abstract

**Background:**

Climbing is an intricate sport composed of various disciplines, holds, styles, distances between holds, and levels of difficulty. In highly skilled climbers the potential for further strength-specific adaptations to increase performance may be marginal in elite climbers. With an eye on the upcoming 2024 Paris Olympics, more climbers are trying to maximize performance and improve training strategies. The relationships between muscular strength and climbing performance, as well as the role of strength in injury prevention, remain to be fully elucidated. This narrative review seeks to discuss the current literature regarding the effect of resistance training in improving maximal strength, muscle hypertrophy, muscular power, and local muscular endurance on climbing performance, and as a strategy to prevent injuries.

**Main Body:**

Since sport climbing requires exerting forces against gravity to maintain grip and move the body along the route, it is generally accepted that a climber`s absolute and relative muscular strength are important for climbing performance. Performance characteristics of forearm flexor muscles (hang-time on ledge, force output, rate of force development, and oxidative capacity) discriminate between climbing performance level, climbing styles, and between climbers and non-climbers. Strength of the hand and wrist flexors, shoulders and upper limbs has gained much attention in the scientific literature, and it has been suggested that both general and specific strength training should be part of a climber`s training program. Furthermore, the ability to generate sub-maximal force in different work-rest ratios has proved useful, in examining finger flexor endurance capacity while trying to mimic real-world climbing demands. Importantly, fingers and shoulders are the most frequent injury locations in climbing. Due to the high mechanical stress and load on the finger flexors, fingerboard and campus board training should be limited in lower-graded climbers. Coaches should address, acknowledge, and screen for amenorrhea and disordered eating in climbers.

**Conclusion:**

Structured low-volume high-resistance training, twice per week hanging from small ledges or a fingerboard, is a feasible approach for climbers. The current injury prevention training aims to increase the level of performance through building tolerance to performance-relevant load exposure and promoting this approach in the climbing field.

## Key Points

- Altering the mechanical and metabolic stress, by using different intensities or varying the number of repetitions and sets, and training frequency per week are the most significant variables in manipulating the overall training volume and the variables requiring emphasis in planning incorporation of resistance training (RT) in climbers.
- Structured low-volume training at high resistance, twice per week, is a feasible approach to RT in climbers.
- The following classifications are proposed in climbing: > 15 reps (or hang time > 30 s) strength endurance bias; 8–15 RM (or 3–30 s hang time) hypertrophic bias; 1- 5 RM (or 1–5 s hang time) maximal strength bias.
- Improving maximal finger and shoulder girdle strength may decrease injury risk; as a result of reducing percentage of maximum strength generated in each move, the overall loading stress in a session is also diminished.
- Upper body RT programs have proven efficient for improving performance in climbing-specific tests among lower- and intermediate-grade climbers, but whether this training approach may improve climbing performance among advanced or elite climbers remains to be elucidated.

## Background

Climbing is an intricate sport composed of various disciplines, holds, styles, distances between holds, and levels of difficulty [1]. Climbing performance is not solely limited by absolute or relative strength of the fingers and upper body, but is largely determined by an interaction of a climber`s abilities [2, 3] endurance (i.e., measured by hanging time on a ledge) [4, 5], strength (i.e., maximal isometric force output on climbing holds) [6, 7], and flexibility (e.g., leg abduction or range of motion of the arms) [8–10]. Muscles which flex the fingers have a key role in climbing and these muscles in climbers are shown to have higher oxidative capacity index, relative strength (strength-to-weight-ratio), and overall strength level in climbers, compared with non-climbers [11]. Furthermore, performance characteristics of forearm flexor muscles discriminate between climbing performance level [12–14]. An overview of workload demands in climbing reveals the sport requires diverse physiological capacities, based on the combination of volume (duration, route length, number of moves), intensity (movement velocity, % of maximal force applied on hand- and foot-holds, wall inclination) and work-rest parameters (contact times with the holds and work-to-rest ratio) [3]. For example, a recent study including 73 climbers, with performance levels ranging from intermediate to advanced, concluded that number of consecutive pull-ups and bent-arm hang duration were the strongest predictors of climbing performance [2]. Similarly, Balas et al. [12] demonstrated that finger hang time to failure on a 25 mm rung was the strongest predictor of climbing performance. Of note, climbing performance is most frequently reported as the best red-point (i.e., successfully climbed a practiced route) or on-sight lead (i.e., successfully climbed a route without any prior knowledge about the route) or bouldering performance in the last 3–12 months [12, 15] which has proven to be a valid and accurate reflection of climbing ability [16]. More recently, differences in climbing-specific strength attributes between boulderers and lead climbers have been demonstrated [17, 18]. Specifically, boulderers exhibited greater force output (i.e., maximal isometric voluntary contraction (MVIC) using an open crimp grip) and were more explosive (i.e., higher rate of force development (RFD) and pull-up velocity) compared to lead climbers, whereas similar finger flexor endurance was observed between the groups [17, 18]. Lead climbers demonstrated greater oxygen capacity and recovery kinetics in muscles of the finger flexor compared with boulderers [19, 20]. High levels of local muscle oxidative capacity in climbers were expressed as increased finger flexor muscle de-oxygenation (oxygen extraction end use) during isometric muscle contractions, and re-oxygenation during short relaxation phases [19, 21–23]. In addition, recent studies showed that maintaining maximal finger flexor force during 30 s all-out test or intermediate contraction to fatigue using a climbing specific hold are more important than muscle aerobic capacity in higher elite climbers [24].

Both finger flexor strength and finger flexor endurance on climbing holds have been suggested as crucial factors for predicting climbing performance [2, 12] and in addition, discriminate between climbers at different levels [25], as well as climbers from non-climbers [26]. For instance, more advanced climbers exhibit greater finger flexor critical force (i.e., the percentage of maximal force that can be sustained over an extended period) and higher levels of de- and re-oxygenation during hangboard tests compared to less advanced climbers [19, 27]. Similarly, during progressive climbing with increased speed or steeper wall angles, climbers of higher ability demonstrate longer time until exhaustion [28, 29], which was associated with a slower decrease in forearm muscle oxygen saturation and a shift in the localized metabolic threshold (corresponding to critical power/force) to a higher intensity than in their less able counterparts [30]. Aerobic capacity at the systemic level plays a substantial role during climbing, although it does not seem to discriminate between climbers of different ability levels [31, 32]. For example, aerobic and work capacity, traditionally assessed through maximal incremental running or cycling tests, do not correlate with climbing ability [32, 33] or distinguish climbers from non-climbers [23]. However, climbing at sub-maximal intensity at a comfortable speed may require up to 70% of VO_2_ peak determined on cycloergometer or treadmill showing the important contribution of systemic aerobic capacity during climbing. [1, 5, 32]. Thus, climbers should use training methods which reflect climbing specificity. It is worth noting that resistance training (RT) for climbers may derive from traditional RT in a gym lifting external loads [34]. Typically, RT for climbing is conducted hanging from small rungs or a fingerboard [35–37], or performing a low number (e.g., 4–8) of bouldering moves at high-intensity [18, 38]. In a recent review [34], different RT approaches in climbing were classified as non-specific (traditional resistance exercises, without kinematic or dynamic similarities to the climbing movement, targeting the upper body and arms), semi-specific (with high kinematic or dynamic similarity to the climbing movement, e.g., finger hang, fingerboard, campus board training), and specific for climbing (bouldering and lead climbing). Furthermore, muscle contraction intensity, number of repetitions, or duration of the exercises, were used to categorize the training methods as strength endurance, hypertrophy, or maximal strength [34]. Upper body RT programs have improved performance in climbing-specific tests among lower- and intermediate-grade climbers [39], but whether this training approach may improve climbing performance among advanced or elite climbers remains to be elucidated. Consequently, this narrative review seeks to discuss the current literature regarding the effect of RT in improving maximal strength, muscle hypertrophy, power, and local muscular endurance on climbing performance, and as a strategy to prevent injuries.

To summarize, climbing performance is largely determined by an interaction of multiple physiological and mental abilities. Still, performance characteristics of forearm flexor muscles (hang-time on ledge, force output, RFD, and oxidative capacity) discriminate between climbing performance level, climbing styles, and between climbers and non-climbers. Typically, these muscles are trained climbing specifically using small ledges or a fingerboard with high intensity and few repetitions.

## Main Text

### Resistance Training in Climbing

RT is the most efficient approach to increase skeletal muscle mass, strength, and power [40, 41]. It may also increase muscle mitochondrial content [42, 43] as well as cardiorespiratory fitness [44], although only a limited number of RT interventions with climbers as participants have been conducted [34, 45]. However, to improve climbing performance, climbers emphasize maximal relative strength and power in the shoulder girdle and back muscles [34, 45]. Therefore, despite the limited number of RT interventions conducted with climbers as participants [34, 45], evidence from the general literature on RT could be transposed to the context of climbing conditions. For example, several studies have indicated that completion of strength training programs leads to an increase in absolute strength and sub-maximal force generation capacity in climbers [36, 37, 46, 47]. In support of this approach, a recent review of climbing classified > 15 repetitions (or hang time > 30 s) as strength endurance, 8–15 RM (or 3–30 s hang time) as hypertrophy training, and 1–5 RM (or 1–5 s hang time) as maximal strength training [34]. To identify potential papers examining climbers, we conducted two systematic searches. The systematic searches were based on recently published meta-analyses [34, 45] and reviews in climbing [48, 49]. Peer-reviewed articles in English from the databases SPORTDiscus, SCOPUS, PubMed, and Google Scholar were used with search terms (“rock climb*” OR “sport climb*” OR “lead climb*” OR “climbers*” or boulder*”) AND (“forearm strength”* OR “forearm endurance*” OR “finger strength*” OR “finger endurance*” OR “finger flexors*” OR “campus board*” OR “fingerboard*” OR “hangboard*” AND “finger injuries*” OR “injuries climbing*” OR “injuries bouldering*” AND “testing*” OR “RFD*”, “power*” OR “performance*. The main search was conducted on November 13th, 2021, and repeated on November 4th, 2022.

Climbers are attempting to generate enough force and friction on the holds to avoid losing contact with the climbing surface, while maintaining static positions or accelerating their body mass upward. The importance and effectiveness of RT may be explained by Newton`s second law of motion (⅀ forces acting on an object = object`s mass x object`s acceleration). According to this law, the change in motion of an object (e.g., the climber`s acceleration) is directly proportional to the force applied. In the context of climbing, greater force generation, producing greater acceleration, could enable a climber to reach further to grasp a hold. However, as gravitational forces affect the mass of climbers, greater force is needed to complete a move with greater body mass. Therefore, muscle hypertrophy associated with RT may result in an undesired effect for a climber, as greater cross-sectional area (CSA) associated with hypertrophy could influence body mass and increase it. Typically, high level climbers aim for high relative strength (especially in the shoulder girdle and fingers) and increased muscle mass, as a result of greater muscle CSA, which implies more sarcomeres in parallel and/or contractile filaments to generate force [50]. Therefore, while there may be a ‘trade-off’ with the potential effect on whole-body mass, up to a certain point, increased muscle CSA should be beneficial to improve climbing performance.

To summarize, RT in climbing is conducted in traditional gyms but with climbing specific equipment like fingerboards or campus board. To translate traditional RT approaches into climbing, > 15 repetitions (or hang time > 30 s) was classified as strength endurance, 8–15 RM (or 3–30 s hang time) as hypertrophy training, and 1–5 RM (or 1–5 s hang time) as maximal strength training.

### Assessment of Strength in Climbing

Regular assessments of athletes’ training status, mental and physical health, muscular soreness, and performance levels have been standard practice in many sports for several decades but have barely been discussed in the climbing literature. Regarding testing and monitoring in climbing, a variety of tests have been introduced [49, 51]. Currently, there are no established scales that recommend a standardized level of strength for climbers of varying performance levels or describing potential sex-based differences. In more explosive sports, it has been suggested that athletes who are able to back squat at least twice their body mass, to a depth of thighs parallel to the floor, or to 90° of knee flexion [52–54], can jump higher [55], sprint faster [56], and achieve muscle potentiation earlier [53, 57] than individuals with lower squat strength capacity. These findings have been implemented in a model that distinguishes three primary strength phases: the strength deficit phase, the strength association phase, and the strength reserve phase [54, 58]. For high performance climbers, the last phase may be the standard to aim for. At this stage, an athlete has improved their ability to produce force, primarily as a result of adaptations (central and local) and through skill acquisition in specific tasks. Despite the potential for further enhancement of relative strength, the benefit to overall performance may be less pronounced than in preceding phases. In fact, it has been suggested that athletes may experience a negative effect on strength performance if they maintain a very high level of strength [59], leading to the recommendation to prioritize power or explosive training, once a certain threshold of strength is achieved [59]. Typically, improving strength capacity in climbing is emphasized in the off-season, with a shift toward training power and explosive capacities closer to the competition period. Moreover, it could be speculated that if climbers have achieved a certain threshold in the finger hang or dead hang test (> 60 s, using a shallow rung), the improvement in climbing performance following further increases in dead hang duration may be reduced, compared to the potential for increase in lower-grade climbers. This phenomenon of a threshold in improvement may explain the association observed between these tests and climbing performance in the literature [2, 12]. Accordingly, there is a need to establish valid and reliable climbing-specific tests [49, 51] to determine the relative effectiveness of different training approaches in improving performance at elite level.

For example, greater maximal strength in the finger flexors and shoulder girdle has been linked to higher performance outcomes in climbing [34, 39, 45]. Still, there is an on-going debate regarding the transferability of general strength to a sport-specific task (e.g., leg strength training for improving jumping or sprinting) [60]. As the transferability of strength gains into improvement in force–time characteristics is viewed as a positive adaptation, strength in relation to the required sports-specific skills and performance demands is paramount. For example, if the strength characteristics of an athlete do not transfer into performance gains in a given sport, coaches may be less interested in incorporating RT as a method of preparing their athletes to perform. It`s generally accepted that improved maximal muscular strength can alter peak force output and the shape of the force-curve [61–63] resulting in a greater net impulse, which may be a determinant of individual performance [64]. Despite this evidence, only a limited number of studies with climbers have been conducted on improving general and climbing-specific strength, and studies have only included intermediate to elite level climbers [34, 45]. Importantly, transferability of strength gains is likely greatest when tasks mimic the RT intervention (e.g., muscle groups involved and contraction forms). [65, 66]. For example, strength gains may be expected to be greater in dynamic muscle tasks (e.g., pull-up strength) than in isometric tasks (e.g., bent-arm hang time with external loads or maximal isometric pull-up tests) after a dynamic upper body resistance program. As highlighted in a recent paper addressing the concept of functional training [67], the phrase “You`re only as strong as your weakest link” perfectly describes the interaction of different physiological skills required for successful climbing performance. More specifically, a climber may be able to perform many pull-ups, but may not use this capacity fully if their finger flexor strength is underdeveloped and the climber cannot maintain contact with the holds. Therefore, task transferability between different rungs/holds and grip positions (pinch, crimp, open hand) needs to be examined when designing finger- and grip strength training programs [68], and the transferable impact of specific training considered in relation to overall climbing performance.

To summarize, a variety of different climbing specific tests have been conducted to examine different capacities. Several of these tests discriminate between climbing performance level and climbing styles and have proven valid and reliable. Still, only a handful of studies have included outcomes of climbing performance (i.e., lead or bouldering performance). The transferability of improving climbing specific test performance to actual climbing performance needs to be examined.

### Adaptations to Resistance Training for Climbers

Both mechanical load and metabolic stress are important factors/stimuli to consider in RT [69], especially when starting a new training approach, such as prescribing specific finger training in climbers, and should be considered against overall training volume, which may already be high in elite climbers. Training intensity (% of maximum force output, repetition maximum (RM), or time under tension) [34, 54], training volume (numbers of sets per muscle group) [70], and training frequency (sessions per week) [71] are the most frequently used training variables to periodize stimuli to specifically target muscle strength, muscle hypertrophy, or local muscle endurance capacity [72]. Fingerboard training is one of the most frequently used training devices in climbing. Fingerboards contain different holds (jugs, edges, or slopers), sizes (one or four fingers’ pockets) and depths (generally 8–35 mm). Usually, climbers hang vertically, holding on to the fingerboard with different intensity (body weight, body weight + extra loads or edge depth) conducting either isometric (e.g., dead hang) or dynamic contractions (e.g., pull-up). High mechanical loads, such as high-intensity short-term fingerboard training (e.g., two finger holds with 3–4 min inter-set pauses), appear to be important for muscle strength, whereas metabolic stress caused by higher training volume (e.g., multiple repetitions and sets with short inter-set pauses (1–2 min)) appears more important for muscle hypertrophy [3, 34]. Regarding intensity, “lighter” loads/intensity have been referred to in the general RT literature as < 60% of max (or 15–25 RM), “moderate” loads/intensity as 60–85% of max (6–12 RM), and “heavy” loads/intensity as > 85% of max (1–6 RM) [72–74]. However, these categories are dependent on training status, exercise type, and muscle(s) involved [72]. Furthermore, lighter loads with 15–25 RM have been suggested to improve muscular endurance, whereas both moderate and heavy loads (1–12 RM) improve muscular strength and hypertrophy [72]. Therefore, it is generally considered that adaptations following RT in general and in climbing are load dependent. However, an emerging body of evidence suggests that muscular strength and hypertrophy occur across a spectrum of very light to heavy loads, as long as the training is conducted with a high level of effort and at maximal intended velocity [39, 73–75]. More recently, 6 weeks of general RT using 30% of 1-RM to failure increased maximal strength by 9.5% among recreationally active men with no RT experience over the previous six months [76]. Importantly, neural adaptions including enhanced motor unit recruitment, increased firing frequency, enhanced muscle activation, and improved inter- and intra- muscle coordination [77, 78] contribute to strength improvements [77]. Increased muscle strength may be achieved by improving the contractile filaments within the muscles, or by increasing cross-sectional area by enlarging the size of each muscle fiber [77].

Block periodization (e.g., training periods of five weeks prioritizing one physical attribute, while maintaining others) may be an effective way to counteract the phenomenon of adaptive attenuation and improve specific capacity [36, 37], as most strength improvements occur in the initial weeks of training [79, 80]. It has been speculated that higher loads cause greater improvements in muscle function than lower loads [76] since high-load, low-volume RT programs, conducted at maximal velocity [81], facilitate neural adaptions and improve muscle strength, independently of muscle hypertrophy [82]. Furthermore, the dose–response relationship between both training volume and muscle hypertrophy [74, 83], and between training volume and strength [84], have been examined in the general RT literature. However, and with special interest for climbers, the concept of “the minimal effective training dose” required to increase maximal dynamic strength has been introduced in the general RT literature. Typically, a single set of 6–12 repetitions performed to failure, with loads ranging from 70 to 85% of maximum, performed 2–3 times per week for 8–12 weeks, can produce a suboptimal, but significant increase in strength [72, 85, 86]. For climbing specific finger training, four weeks (3 times per week) of fingerboard training (6 grip exercises, 2 series of 4–6 s effort) improved maximal force in elite and top world-ranking climbers [37]. For lower-level climbers (advanced and elite level), brief but high-intensity campus board training (2 sessions per week, 4 exercises, 4 sets) over four weeks improved bouldering performance [36].

In summary, a limited number of RT interventions have been conducted in climbing. At the present time, much of the evidence of morphological and neurological adaptations is generated from the general RT literature and transposed to the context of climbing conditions.

### Resistance Training for Increasing Muscle Hypertrophy for Climbers

The nature of climbing causes a metabolic and mechanical stress on the forearms, finger flexors, and upper body. Typically and independent of climber moves or general RT exercises, moderate intensity RT approaches (i.e., 60–85% of max load or 6–12 RM) focusing on metabolic stress have been found to lead to a greater increase in muscle CSA than approaches emphasizing mechanical loading (90–100% of maximum load or 1–3 RM) [87, 88]. In general, a minimal intensity of 60% of max is necessary to elicit significant hypertrophic adaptions, as a minimal threshold stimulus is required to activate the largest motor units and thereby the different muscle fiber types [75, 89, 90]. The American College of Sports Medicine has suggested that lighter loads promote greater endurance adaptions, whereas heavy loads promote greater strength adaptions [72]. Of note, the potentially counterproductive outcome of greater muscle hypertrophy (i.e., greater muscle CSA), is an increase in muscle mass. Furthermore, a massive increase in fiber CSA could potentially reduce capillary density to muscle fiber cross-sectional ratio if not compensated for [91]. In the context of climbing, increased CSA may reduce lead climbing performance due to reduced endurance capacity if muscle fiber capacity is not maintained within the same training period. Importantly, climbers aiming to improve lead climbing performance should therefore include low- to moderate lead climbing sessions in the same training period focusing on improving CSA whereas the muscle fiber endurance capacity may be less important in bouldering [3, 68]. In a meta-analysis, Schoenfeld et al. [74] stratified gains in muscle hypertrophy based on sets conducted per week in traditional RT exercises (< 5 sets, 5–9 sets, and > 10 sets) and reported increases of ~ 5%,  ~ 7%, and ~ 10%, respectively. Based on the findings of Schoenfeld and colleagues [74], it could be argued that the weekly number of sets is a significant factor in improving muscle hypertrophy.

Several coaches and climbers avoid high-volume RT due to fatigue, a reduction in specific climbing training, and fear of increased body mass due to a higher muscle volume. Brief, structured, and low-volume (e.g., one- to two sets) of twice weekly, high-intensity RT may be a feasible approach for climbers [36]. In general RT, a meta-analysis concluded that conducting multiple sets was associated with 40% greater hypertrophy-related effect size than 1 set, in both untrained and trained subjects [70]. Nevertheless, no significant difference in muscle hypertrophy was observed between performing 2–3 sets and 4–6 sets per exercise, suggesting that the effect of increasing from 1 set to 2–3 was greater than increasing from 2 to 3 sets to 4–6 [70]. However, block periodization (e.g., training periods of five weeks prioritizing one physical attribute, while maintaining others) may be an effective way to counteract the phenomenon of adaptive attenuation and improve specific capacity [36, 37], as most strength improvements occur in the initial weeks of training [79, 80]. It has been speculated that higher loads cause greater improvements in muscle function than lower loads [76] since high-load, low-volume RT programs, conducted at maximal velocity [81], facilitate neural adaptions and improve muscle strength, independently of muscle hypertrophy [82].

Translating these findings to climbing, RT with lighter loads may be recommended in lead climbing, whereas heavy loads may be a more ecologically relevant stimulus in bouldering. Lead climbing is performed on walls higher than 10 m and comprises multiple climbing moves, whereas bouldering consists of eight-to ten moves on a shorter wall (< 5 m) and is performed without ropes [17, 92, 93]. Mitchell et al. [89] compared maximal strength, muscle hypertrophy, and repetitions to failure in recreationally active men, using loads at both 30% of 1RM and 80% of 1RM, after a heavy (80% of max) or light (30% of max) knee extension training program. The intensities (i.e., 30% and 80%) are not directly comparable to lead climbing and bouldering, but the number of repetitions performed at each intensity corresponds to the numbers of actions in lead climbing competitions (i.e., several sub-maximal intensity moves lasting between 2 and 6 min) [93] and bouldering competitions (5–10 powerful moves) [92]. Interestingly, no between-groups differences were observed, but both training conditions demonstrated improvements in muscle volume and maximal strength. Furthermore, there were distinct, task-specific adaptations under both conditions, as only the 80% training demonstrated an increase post-intervention in numbers of repetitions using heavy loads (e.g., 80% of max), whereas only the 30% group increased in numbers of repetitions performed at lighter loads (e.g., 30% of max) [89]. In the context of climbing, evidence from this study suggests that RT intensity could be manipulated depending on whether a climber is aiming to improve the lead climbing (i.e., local endurance capacity) or bouldering (i.e., conducting few but high intensity moves). In the 2024 Paris Olympics, climbing will be divided into three disciplines (lead, bouldering, and speed) and not the combined approach (the combined score of each discipline) used during the 2021 Tokyo Olympics. Most likely, greater specialization in one climbing discipline will emphasize training approaches to improve task-specific adaptations which means that lead climbers could emphasize to a greater extent using multiple repetitions with low-to moderate intensity, whereas boulderers could emphasize high intensity but few repetitions RT.

More recently, strength and conditioning researchers have suggested that the same adaptions are possible to achieve without training with heavy loads (> 65% of max), using lighter loads (< 60% of max) but continuing lifting to momentary failure [94, 95]. Of note and in relation to this proposal, low-intensity RT (< 60% of max) performed with blood flow restriction (BFR) can promote an increased level of metabolic stress, resulting in significant muscle hypertrophy [96]. BFR has been used in rehabilitation to decrease mechanical loading, while generating a similar metabolic stress as high-intensity RT [97]. In climbing, only one cross-sectional study has examined the effects of BFR on climbing-specific tests, targeting finger flexor maximal strength and endurance capacity [15]. The authors demonstrated similar results, comparing the tests with or without BFR [15]. Recently, a five week low-load BFR training (30% of max) twice per week maintained isometric finger flexor strength and endurance in climbers [98]. Since climbing necessarily restricts or reduces upper body blood flow (arms over head, high-intensity finger flexor activation), training interventions using BFR to enhance performance (e.g., improve muscle hypertrophy and/or strength), or to reduce the mechanical stress on the fingers (e.g., result in greater climbing volume), should be examined.

To summarize, general RT with moderate intensity (i.e., 60–85% of max load or 6–12 RM) increases metabolic stress and causes muscle hypertrophy whereas mechanical load (90–100% of maximum load or 1–3 RM) emphasizes maximal strength. Both muscle hypertrophy and strength can be improved using the same principles as for general RT targeting climbing related muscles (shoulder girdle, arms and fingers) and exercises (fingerboard, campus board and holds).

### Resistance Training for Increasing Maximal Strength for Climbers

One of the most extensively studied relationships in climbing is the capacity to develop maximal strength after a RT period [45]. When training to momentary failure, the current RT literature has demonstrated that both light (< 60% of max) and heavy (> 65% of max) loads can effectively improve strength [74]. However, training with heavier loads (e.g., 8–12 repetitions vs. 25–35 repetitions) without reaching momentary failure may still produce notable enhancements in maximal strength, while concurrently minimizing recovery time [74, 99]. Several studies including different populations have demonstrated the efficacy of low-volume traditional gym facilitated RT programs with high to maximal intensity for, on improving muscle strength, compared to higher RT volumes at lower intensity [66, 100, 101]. Low-volume but high-intensity RT may be a feasible approach to increase general force capacity (i.e., pull-up strength) and specific strength (i.e., finger grip strength) in climbers, especially boulderers [37, 92]. For example, Izquierdo et al. [102] showed similar increases in muscle strength when comparing training to failure (3 sets of 10-RM) to sub-maximal training (6 sets of 3–5 repetitions) at the same training intensity (75% of 1-RM). [73]. In a meta-analysis, Ralston et al. [103] compared maximal strength improvements in response to low (1 day per week), medium (2 days per week), or high (≥ 3 days per week) resistance training frequency. When training volume (e.g., numbers of sets x repetitions) or volume loading (e.g., sets × repetitions × loads) were matched, no significant difference in training frequency was observed with 1-RM [103]. In fact, performing a medium (5–9) or high (≥ 10) number of sets of strength training for each muscle group is shown to be marginally more effective than a lower (≤ 5) number to optimize strength improvement [71]. This observation further supports the concept of micro dosing to achieve a ‘minimal effective dose’ within athlete training, reducing the need for periodized recovery after high-volume single dose exposure. Importantly, the repetitions-to-failure protocol is likely to increase the duration of rest intervals due to eliciting greater fatigue. In bench press, Drinkwater et al. [104] demonstrated 57% longer rest intervals between sets following 4 sets of 6 repetitions to failure (260 s), compared to 8 sets of 3 repetitions not performed to failure (113 s). Importantly, both “low-volume—high-load” and “high-volume—low-load” RT can improve muscle strength in untrained and resistance trained individuals [65, 100]. Nevertheless, it is likely that these training approaches may become less effective under chronic exposure, as strength levels improve over time [79]. Still, only one study in low level climbers has compared upper body RT with “high repetitions—low loads” and “few repetitions—high loads”. The training produced improvements in both intervention groups, with no differences observed in dead hang duration, pull-up strength [39] or, more interestingly, in climbing performance (lead climbing using competition format), between the two intervention groups [39].

Regarding climbing, Levernier and Laffaye [37] trained national and international competitive boulderers three times per week. The training consisted of six finger grip exercises (2 sets of 4–6 s contraction, 3 min rest) using the slope and half crimp grips and demonstrated an 8% improvement in maximal slope crimp strength after four weeks. At post-test there was no significant difference between the training groups in maximal force compared to the control group performing climbing exercises only. Training on a campus board is one of the most climbing-specific finger strength approaches in climbing and involves a series of dynamic moves on shallow rungs (< 30 mm) targeting the entire pulling apparatus, without assistance from the feet. Stien et al. [36] examined the effects of a five-week campus board training-block in advanced- to elite-level climbers and demonstrated improvement in maximal finger strength, bouldering performance, maximal reach and number of campus board moves to failure, compared with maintaining usual climbing routines. A potentially effective method of avoiding training to failure is to monitor the training intensity using repetitions in reserve (RIR) [54]. The RIR has been used to avoid the acute neuromuscular fatigue and negative perceptual responses [105]. For example, Refalo et al. [105] demonstrated a greater decrease in barbell velocity in bench press in resistance trained subjects after conducting sets to failure (− 25%), versus 1-RIR (− 13%) and 3-RIR (− 8%). In isometric fingerboard training this could be applied by using seconds in reserve (e.g., 2 s in reserve after 10 s of work) in order to expose climbers to progressively higher intensity actions yet avoid the need for time off climbing for periodized recovery.

Stien et al. [36] compared two vs. four weekly campus board training session on bouldering performance and maximal isometric pull-up strength among advanced-to-elite climbers. Both groups conducted similar numbers of weekly training sets. Compared to a control group (continued climbing as usually), four weekly campus board sessions improved maximal force and RFD whereas two weekly campus board improved bouldering performance and maximal moves to failure on the campus board. No differences were observed between the two campus board groups which might have been due to a short training period (5 weeks), low campus board training volume (four exercises conducted four times) and few climbers in each group (*n* = 5 and *n* = 6) [36]. To the author's best knowledge, Stien et al. [36] is the only study examining weekly training frequency in climbing. However, in the strength and conditioning literature the effects of different session distributions with the same overall volume have been investigated. For example, increasing training frequency has been proposed as a method to increase intensity during training sessions [106]. In the general resistance training literature, it has been shown that distributing the same number of sets across two shorter sessions per day led to greater overall training volume [107], increased maximal strength [108], higher testosterone levels [109], and increased neuromuscular activation, compared to a single long session [88]. Two shorter sessions were also perceived as less uncomfortable and demanding lower exertion than one long session [107]. However, a higher training frequency was favorable compared to lower weekly frequency for the upper body [103]. Still, there is a rationale for increasing weekly training frequency in order to increase overall training volume. For example, Gonzales-Badillo et al. [110] concluded that moderate training volumes were superior for increasing maximal strength in weightlifting exercises compared to lower- and higher volumes among Olympic weightlifters, which suggests strength gains are achievable if volume of exposure is sufficient to promote adaptation. Whether these findings using multiple joint exercises and targeting lower-body muscle groups are transferable to smaller muscles groups (i.e., finger flexor for climbers) remains to be proven. More recently, a meta-analysis [111] compared low vs. high weekly training frequency (ranging from 1 vs. 2 sessions to 3 vs. 9 sessions) in well-trained populations of team sports athletes, competing up to three times per week. The authors concluded that over a 6–12 week period, no clear differences in maximal strength development were observed for the different training frequencies studied. Instead, the authors recommended low volume but frequent training exposure, distributed across the week, rather than condensing the training into focused sessions, once or twice per week [111].

Bouldering can be viewed as a form of specific strength and power training for climbers [34]. Bouldering is performed on lower walls and often comprises few, but highly explosive and challenging moves [92]. Accordingly, fingerboard or campus board training have proved effective for improving maximal finger strength and mimic bouldering regarding moves and the intensity of the moves [35, 36]. Furthermore, four weeks of finger grip training in elite- and top world-ranking climbers improved rate of force development and maximal force [22]. Furthermore, dead hang performance was improved following a generic program of upper body strength training, using either high-loads and few repetitions or low-loads and high repetitions [37, 39]. Despite empirical evidence suggesting a correlation between climbing-specific and general strength and climbing performance, there is yet to be conclusive proof of a causal relationship. Further research is needed to establish the extent to which specific isometric (e.g., fingerboard strength), specific dynamic (e.g., campus board moves), and/or general strength (e.g., upper body strength or pull-up capacity) contribute to improved climbing performance. Notably, Medernach et al. [46] demonstrated interval bouldering (4, 6, 8, and 10 moves with no rest between moves, 4 sets, 7 min rest between sets) improved intermittent finger hangs (8 s hang time, 4 s rest on a 30 mm deep fingerboard) and climbing time to exhaustion among highly advanced competitive boulderers. Further studies examining the relationship between climbing-specific performance outcomes and prescribed exposures to training movements at varying volumes and frequencies are needed.

The interference effect of combining RT (i.e., high-intensity bouldering) with endurance (i.e., lead climbing or low-load high repetitions resistance program) is frequently discussed in the scientific literature [91, 112]. Combining both strength- and endurance training has often been referred to as concurrent training [112]. Moreover, the fact that some athletes in the World Cup are successful in two disciplines (bouldering and lead climbing) could indicate that the two disciplines do not represent as different demands as previously assumed [113]. But given the different competition styles (4–10 explosive moves vs. 30–50 moves), different physical requirements may separate these climbing styles [17, 18]. Still, and based on previous literature combining strength training and endurance training, a 3–6% increase in muscle hypertrophy of the main target muscles has been observed [114, 115]. It should however be noted that an ‘interference effect’ on maximal strength development has been reported in some [116], but not all [112] studies.

In summary, climbers aim to improve relative strength using climbing specific approaches. Maximal strength measures with climbing specific tests distinguish climbing performance and climbing style. Low-volume, high intensity RT involving fingerboards, campus boards, different holds and grips are feasible approaches to improve the maximal strength capacity and may be used as a supplement to climbing training. Still, longitudinal high-quality studies are needed.

### Resistance Training for Increasing Power Output and Rate of Force Development in Climbers

The capacity for powerful movements is essential in competitive climbers. Despite this, mechanical power (e.g., product of force x velocity) is barely addressed in the climbing literature [36, 117–121]. Draper et al. [118] demonstrated elite climbers produce superior arm power compared to novices during arm-jump exercises. Furthermore, Vigouroux et al. [119] examined the influence of different grip conditions (10–22 mm holds) on maximal power in pull-up and found significantly lower power output using the 10 mm hold, compared to the larger sizes. This latter study showed that grip strength may represent the limiting factor determining ability to generate power, which perhaps explains why many climbers emphasize maximal finger flexor strength in their training routines [3, 13]. Elsewhere in the literature, Levernier et al. [121] reported that power output in pull-ups was different between climbing disciplines (i.e., bouldering, lead- and speed- climbers) in higher-elite athletes, which supports the proposal that upper body power is a significant factor in elite climbing performance. Interestingly, boulderers demonstrated greater power than lead- and speed climbers, suggesting a higher requirement for power may be a feature of this discipline [121]. However, as limited training studies examining power have been conducted, conclusive evidence into different power profiles according to climbing event is lacking. Stien et al. [36] examined the effects of campus board training frequency (2 vs. 4 session per week vs. control) and demonstrated significant improvement in power, measured as maximal reach, in advanced and elite climbers after five weeks of training. There was no difference between the intervention groups; however, both intervention groups improved more than the control group. To the authors’ best knowledge, no training intervention has examined the effects of a power training program on climbing performance specifically.

Climbing studies including measurements of rate of force development (RFD) have increased in the last decade [49]. Most recently, Vereide and colleagues [14] demonstrated 154% and 75% greater RFD in a 23 mm campus rung isometric pull-up exercise in elite climbers (IRCRA score 24–27) compared to intermediate (IRCRA score 10–17) and advanced climbers (IRCRA score 18–23). Independent of performance level, the authors also reported a moderate association (*r* = 0.65) between RFD and climbing performance. Stien and colleagues [122] demonstrated that maximal force and RFD can distinguish between climbing performance levels, and that elite climbers were superior in maximal force and RFD, compared to advanced- and intermediate climbers. Additionally, Levernier and Laffaye [37] demonstrated 25–33% improvement in RFD for the slope, half- and full crimp after four weeks of fingerboard training in elite, top-ranked climbers. Furthermore, ten weeks of isometric (7:3 s work/rest ratio) fingerboard training resulted in 39% improvement in RFD (ES = 0.86) in intermediate- to advanced level climbers, but not to a greater extent than the effect of continuing regular climbing training routines (*p* = 0.056, ES = 0.21) [35]. Of note, the fingerboard training did not focus on rapid force generation but prioritized continuous and progressive hang-series (6 series of 7 repetitions (1 repetition being 7 s work and 3 s rest)) which might have resulted in the non-significant difference compared to regular climbing routines [35]. Finally, Stien et al. [36] examined the effects of campus board training and demonstrated 23% improved RFD in a climbing-specific test. It should be noted that explosive training (i.e., using fingerboard, campus board, or different grips), aimed at improving power output or RFD in climbing, requires high-intensity activity, conducted at maximal effort or movement velocity. Therefore, a substantial climbing training history and strength level are required, and power/RFD targeted training should be incorporated carefully in the overall training plan, progressed gradually, and emphasized in different periods throughout the year. In general, high-intensity fingerboard- and campus board training exposure should be limited in beginners and intermediate climbers, due to the potentially high stress applied to the finger flexors.

To summarize, explosive strength distinguishes different climbing performance levels and disciplines. Improving explosive strength in the upper body is a significant factor in elite climbing performance. Due to the high mechanical stress and load on the finger flexors, explosive finger and campus board training should be limited in lower-graded climbers.

### Resistance Training to Develop Local Muscle Endurance in Climbers

Although there is no specific definition of local muscular endurance training, in terms of number of repetitions or intensity (% of 1-RM), high repetition (e.g., > 15 repetition per set) with light loads (e.g., < 60% of 1-RM) has been suggested to improve local muscle endurance capacity [123]. In climbing, endurance capacity in the forearms, finger flexors, and upper-body under low intensity conditions (‘local muscle training’) has been examined [49]. More recently, intermittent contraction of the isometric finger flexor contractions used in climbing holds has been examined at different work to rest ratios (5:5, 7:3, 8:2, 10:3 s) and testing intensities (45–80% of max) [49]. For example, maximal duration of continuous and intermittent (work to rest ratio 8:2) finger flexor muscle contractions at an intensity of 60% MVC were ~ 60 s and 130–150 s, respectively [20, 24]. Critical force while alternating 7 s contraction and 3 s rest phases represented ~ 40% MVC [27]. These are useful guidelines for predicting maximal climbing-specific exercise durations at different intensities above critical force and can be successfully used to assign training load parameters of various interval methods for climbers. Furthermore, time to fatigue, using percentage of maximum force of the finger flexors or during an isometric hold (bent-arm hang or finger hang) has frequently been reported as an indicator of local muscle endurance capacity in climbers [49]. Theoretically, differences in workload characteristics between lead climbing and bouldering should result in different local muscle endurance capacities in climbers of these styles [3, 13, 92]. Lead climbing consists of a minimum 15 m distance on an artificial climbing wall and requires repeated, sub-maximal force generation (maximal climbing time is 6 min), whereas bouldering comprises 4–8 often explosive moves on < 6 m walls [3, 13, 92]. However, there are few studies comparing boulder- and lead climbers, either acutely or longitudinally, and whereas some have not reported differences in local muscle endurance capacity [18, 124], others have [19, 20]. Typically, however, competitive climbers combine bouldering and lead climbing, and climbers have been successful in the World Cup in both climbing styles. Of interest, increased maximal strength has been positively associated with muscular endurance capacity [123]. The relationship between improved maximal strength and exercise efficiency has been demonstrated in other sports, such as cross-country skiing, running, and cycling [91, 125, 126]. It has been suggested that improved endurance performance following increased maximal strength training may be related to i) delayed activation of the less efficient type II fibers, ii) improved neuromuscular efficiency, iii) conversion of fast-twitch type fibers into more fatigue-resistant type IIA fibers, and iv) increased musculotendinous stiffness [91, 114, 115, 127]. There are studies showing a large strength component during tests which mimic hard lead climbing. The predominantly aerobic intermittent finger endurance test (intensity of 60% MVC) required a high portion of a lactic energy (27%) [24, 128]. With the introduction of individual competitive climbing events in the 2024 Paris Olympics, alongside combined events, as in the 2021 Tokyo Olympics, it may be speculated that elite climbers will specialize in one competition form, resulting in the adoption of more specialized training approaches and prioritizing improvement in local muscle endurance capacity.

In summary, high repetitions light loads RT mimics lead climbing and may be used to improve climbing specific muscle endurance capacity. Intermittent isometric finger flexor contraction with different work to rest ratios and intensities are frequently used as training approaches. The number of intervention studies conducted is few and evidence of potential performance improvement in climbing is limited.

### Injury Prevention and Injuries in Climbing

Despite a limited, but growing body of literature on injury prevention and injuries in climbing, acute and chronic injuries in soft and connective tissue and issues related to weight (eating disorder, amenorrhea) are the most examined topics. The rate of injuries is one of the primary concerns in sport. An injury could potentially slow down the training progression in the short or long term, and in some cases, even be career ending. An evaluation of injury and fatality risk in bouldering and indoor climbing found a low injury rate, minor injury severity, and few fatalities [129]. However, more recently, climbing was reported as having the highest injury rate (non-acute injuries) of all Olympic sports [130]. Compared with other sports, the rate of injuries is almost double [131, 132]. An injured climber, who is unable to perform to his/her usual capacities, may re-organize a planned training structure, or in the worst case, abstain from all forms of training for a certain period of time. In these scenarios, introducing new exercises or exercise modalities raises the probability of injury [133] and therefore may not be welcomed by coaches and/or athletes, as it may be viewed as increasing the risk of injury, depending on personal preferences, experience, or training philosophy. Still, an appropriate and progressive prescription of strength training, including a variety of methods, may decrease the overall occurrence of injuries [57, 134, 135]. RT was cited as protective against climbing non-acute injuries in two previous retrospective studies [136, 137]. Although these studies are retrospective in terms of assessing injury, prevention studies in other sports have reported a decrease in injury rate per 1000 exposure hours in collegiate athletes who attended a strength training program [138]. Furthermore, a meta-analysis indicated that strength training reduced sports injuries to less than one-third of previous levels, and suggested regular RT could reduce overuse injuries rates by almost half [135]. Importantly, retrospective data on sport-specific injuries are not reported in a standardized manner, which may have resulted in these different findings of rate of injuries [129–132].

The contemporary view on injury prevention is to prepare the body for the load it will be exposed to during performance. Of great importance for climbers, RT may reduce the number of injuries in tendons by increasing the structural strength of the connective tissue components within a muscle, and at the periosteum [139]. Fingers and shoulders are the most frequent non-acute injury locations reported in climbing [140–142]. Thus, current injury prevention training aims to increase the level of performance through building tolerance to performance-relevant load exposure and promoting this approach in the climbing field could overcome prior reluctance/uncertainty, which may have prevented climbers from engaging in RT.

Increased maximal strength improves the ability to conduct repetitions to failure at sub-maximal loads [46]. Moreover, increased maximal strength results in lower relative effort required to lift a given absolute load. For example, if a person weighing 80 kg increased their maximal capacity in pull-ups from 100 to 120 kg, a pull-up would be reduced from corresponding to 80% to 67% of the maximum capacity (1RM). Translating the levels of effort to climbing moves, theoretically, a climber would experience a decrease in effort if they augmented their maximal strength, without an equivalent increase in body mass. Hence, improving maximal finger- and shoulder girdle strength may also decrease injury risk. Furthermore, as a reduced portion of the maximum force is generated in each move, the overall loading stress in a session is also diminished. It has been suggested that a reduction in training related stress can lead to a decrease in the risk of chronic injury [143]. Consequently, this leads to an increased tolerance to overall training volume and thereby a potentially shorter recovery period, which again has been shown to be important for increased performance [59, 144, 145]. However, differences in injury locations between the sexes have been demonstrated [140, 142] and RT programs need to be designed accordingly. Weekly training volume (i.e., repetitions x sets x training frequency) seems to be the most important factor in improving muscle strength among trained subjects [146]. Moreover, researchers have speculated that an inverted U-shaped relationship exists between training volume and physiological response [147, 148]. It is important to note that combining a large training volume and high-intensity in RT is associated with greater perceptions of discomfort [149], more muscle soreness [99], less enjoyment [150], and a longer recovery time [99, 149]. It should also be noted that conducting high-volume or high-intensity RT may compromise overall climbing volume and potentially increase risk of injury, due to the need for longer recovery.

A general belief is that RT results in muscle hypertrophy and thereby increased muscle mass and body weight. Unfortunately, this seem to have been interpreted by the climbing community as an argument for not implementing RT in their training protocols. Studies have found that high performing climbers are leaner than age matched controls [12, 151]. Of note, lower percent of body fat has been reported among high-level climbers (both men (9.8%) and women (12.2%)) [12]. However, comparing body fat between climbing performance level (e.g., elite to lower-grade), no significant differences were reported [12]. Furthermore, the coefficients of determination (R^2^) demonstrated that body fat percentage was the weakest predictor of climbing performance, compared to outcomes of climbing-specific tests (finger hang, bent-arm hang, grip strength), climbing experience, and climbing meters per week [12]. The evidence suggests that regular practice of sport climbing leads to changes in body composition (e.g., increased muscle mass and decreased body fat), and not that reduction in body fat improves climbing performance. Furthermore, in a recent study it was found that those with either an eating disorder or disordered eating pattern are twice as likely to sustain an injury [140]. Notably, a recent survey examining amenorrhea and eating disorders among female competing climbers found that 18.5% (18 out of 114) reported amenorrhea [140]. Furthermore, those with amenorrhea had a lower body mass index (BMI), and almost twice (22% vs 13%) as many were struggling with disordered eating, compared to those who were eumenorrheic [140]. This study [140] highlights the importance of addressing, acknowledging and screening for the presence of amenorrhea and disordered eating in climbers. Of note, epidemiological retrospective studies on climbing injuries provide inconclusive evidence into potential relationships between body weight and injury risk [152]; further studies are needed to document the influence of menstrual status, injury profile, and disordered eating to assess potential relationships between these factors in climbers.

In summary, interest in injury prevention and injury research is growing. Fingers and shoulders are the most frequent injury locations in climbing. Current injury prevention training aims to increase the level of performance through building tolerance to performance-relevant load exposure (i.e., RT) and promoting this approach in the climbing field. Coaches should address and monitor BMI and screening of amenorrhea and disordered eating in climbers.

## Conclusion

Climbing has been a limited area of research thus far. However, with the exponential growth of scientific papers being published, it is reasonable to hypothesize that knowledge in this field will increase dramatically in the coming decade. It is of paramount importance to convey scientific findings to coaches and climbers. Additionally, reliable and validated testing protocols must be established to assess, compare and monitor athletes' performance. Using these outcomes, an overall training plan, featuring climbing and RT, could be block periodized, aiming to improve muscle hypertrophy (preparation period), maximal strength (start of competition period), power and RFD (championship period), within a climber’s global training program. Strength, both general and climbing-specific, is of great importance in climbing performance. Upper body RT programs have demonstrated improved performance in climbing-specific tests among lower- and intermediately graded climbers, but whether this training approach may improve climbing performance among advanced or elite climbers remains to be elucidated. Instead of having long resistance sessions of high-volume, we recommend brief, structured, high-intensity RT at low-volume, as a feasible approach for climbers, targeting finger flexors, shoulder, and back muscles. For elite level climbers conducting a high climbing volume, but aiming to improve RT volume, we recommend increasing weekly training frequency, instead of adding multiple sets in longer sessions. Low-volume, structured, high-intensity RT sessions reduce the climber`s fatigue state (i.e., shorter recovery period) and may result in greater training quality in the climbing specific session compared to high-volume RT sessions. We advise coaches and athletes to focus on RT aiming at high relative strength based on high maximal strength, and not high relative strength based on low body mass percentage and a low BMI. Climbers and coaches should be aware of the risk of injury, but also the potential of reducing overall injury risk, by implementing RT in a pre-planned and gradually progressive approach (i.e., sets, intensity, and frequency). Furthermore, studies examining the relationship between BMI, eating patterns and menstrual status in climbers are warranted to determine whether leanness in climbers is a factor in strength performance and injury profile.

## Data Availability

Data sharing is not applicable as no datasets were generated and/or analyzed.

## Abbreviations

BMI: Body mass index
CSA: Cross-sectional area
MVC: Maximum voluntary contraction
RFD: Rate of force development
RM: Repetition maximum
RT: Resistance training

## References

[1] Balas J (2014). The relationship between climbing ability and physiological responses to rock climbing. Sci World J.

[2] MacKenzie R (2020). Physical and physiological determinants of rock climbing. Int J Sports Physiol Perform.

[3] Michailov ML (2014). Workload characteristic, performance limiting factors and methods for strength and endurance training in rock climbing. Med Sport.

[4] Michailov ML (2018). Reliability and validity of finger strength and endurance measurements in rock climbing. Res Q Exerc Sport.

[5] Fryer SM (2018). Hemodynamic and cardiorespiratory predictors of sport rock climbing performance. J Strength Cond Res.

[6] Laffaye G, Levernier G, Collin JM (2016). Determinant factors in climbing ability: influence of strength, anthropometry, and neuromuscular fatigue. Scand J Med Sci Sports.

[7] Levernier G, Laffaye G (2021). Rate of force development and maximal force: reliability and difference between non-climbers, skilled and international climbers. Sports Biomech.

[8] Grant S (2001). A comparison of the anthropometric, strength, endurance and flexibility characteristics of female elite and recreational climbers and non-climbers. J Sports Sci.

[9] Grant S (1996). Anthropometric, strength, endurance and flexibility characteristics of elite and recreational climbers. J Sports Sci.

[10] Draper N (2009). Flexibility assessment and the role of flexibility as a determinant of performance in rock climbing. Int J Perform.

[11] Assmann M (2020). Comparison of grip strength in recreational climbers and non-climbing athletes-a cross-sectional study. Int J Environ Res Public Health.

[12] Balas J (2012). Hand-arm strength and endurance as predictors of climbing performance. Eur J Sport Sci.

[13] Saul D (2019). Determinants for success in climbing: a systematic review. J Exerc Sci Fit.

[14] Vereide V (2022). Differences in upper-body peak force and rate of force development in male intermediate, advanced, and elite sport climbers. Front Sports Act Living.

[15] Saeterbakken AH (2020). The effects of acute blood flow restriction on climbing-specific tests. Mov Sport Sci.

[16] Draper N (2011). Self-reported ability assessment in rock climbing. J Sports Sci.

[17] Fanchini M (2013). Differences in climbing-specific strength between boulder and lead rock climbers. J Strength Cond Res.

[18] Stien N (2019). Comparison of climbing-specific strength and endurance between lead and boulder climbers. PLoS ONE.

[19] Fryer S (2017). Differences in forearm strength, endurance, and hemodynamic kinetics between male boulderers and lead rock climbers. Eur J Sport Sci.

[20] Balas J (2016). Active recovery of the finger flexors enhances intermittent handgrip performance in rock climbers. Eur J Sport Sci.

[21] Fryer S (2015). Haemodynamic kinetics and intermittent finger flexor performance in rock climbers. Int J Sports Med.

[22] Philippe M (2012). Climbing-specific finger flexor performance and forearm muscle oxygenation in elite male and female sport climbers. Eur J Appl Physiol.

[23] MacLeod D (2007). Physiological determinants of climbing-specific finger endurance and sport rock climbing performance. J Sports Sci.

[24] Maciejczyk M (2021). Climbing-specific exercise tests: energy system contributions and relationships with sport performance. Front Physiol.

[25] Booth J (1999). Energy cost of sport rock climbing in elite performers. Br J Sports Med.

[26] Sheel AW (2003). Physiological responses to indoor rock-climbing and their relationship to maximal cycle ergometry. Med Sci Sports Exerc.

[27] Giles D (2019). The determination of finger-flexor critical force in rock climbers. Int J Sports Physiol Perform.

[28] Espana-Romero V (2009). Climbing time to exhaustion is a determinant of climbing performance in high-level sport climbers. Eur J Appl Physiol.

[29] Balas J (2021). Isolated finger flexor vs. exhaustive whole-body climbing tests? How to assess endurance in sport climbers?. Eur J Appl Physiol.

[30] Balas J (2021). The estimation of critical angle in climbing as a measure of maximal metabolic steady state. Front Physiol.

[31] Michailov M (2017). Importance of elbow flexor muscle strength and endurance in sport climbing. J Appl Sports Sci.

[32] Rodio A (2008). Physiological adaptation in noncompetitive rock climbers: good for aerobic fitness?. J Strength Cond Res.

[33] Michailov ML (2015). A sport-specific upper-body ergometer test for evaluating submaximal and maximal parameters in elite rock climbers. Int J Sports Physiol Perform.

[34] Langer K, Simon C, Wiemeyer J (2023). Strength training in climbing: a systematic review. J Strength Cond Res.

[35] Hermans E (2022). The effects of 10 weeks hangboard training on climbing specific maximal strength, explosive strength, and finger endurance. Front Sports Act Living.

[36] Stien N (2021). Effects of two vs. four weekly campus board training sessions on bouldering performance and climbing-specific tests in advanced and elite climbers. J Sports Sci Med.

[37] Levernier G, Laffaye G (2019). Four weeks of finger grip training increases the rate of force development and the maximal force in elite and top world-ranking climbers. J Strength Cond Res.

[38] Medernach JP, Kleinoder H, Lotzerich HHH (2015). Effect of interval bouldering on hanging and climbing time to exhausion. Sports Technol.

[39] Hermans E, Andersen V, Saeterbakken AH (2017). The effects of high resistance-few repetitions and low resistance-high repetitions resistance training on climbing performance. Eur J Sport Sci.

[40] Grgic J (2019). Does aerobic training promote the same skeletal muscle hypertrophy as resistance training? A systematic review and meta-analysis. Sports Med.

[41] Farup J (2012). Muscle morphological and strength adaptations to endurance vs. resistance training. J Strength Cond Res.

[42] Groennebaek T, Vissing K (2017). Impact of resistance training on skeletal muscle mitochondrial biogenesis, content, and function. Front Physiol.

[43] Parry HA, Roberts MD, Kavazis AN (2020). Human skeletal muscle mitochondrial adaptations following resistance exercise training. Int J Sports Med.

[44] Frank P (2016). Strength training improves muscle aerobic capacity and glucose tolerance in elderly. Scand J Med Sci Sports.

[45] Stien N (2023). Effects of climbing- and resistance-training on climbing-specific performance: a systematic review and meta-analysis. Biol Sport.

[46] Medernach JP, Kleinoder H, Lotzerich HH (2015). Fingerboard in competitive bouldering: training effects on grip strength and endurance. J Strength Cond Res.

[47] Mundry S (2021). Hangboard training in advanced climbers: a randomized controlled trial. Sci Rep.

[48] Langer K, Simon C, Wiemeyer J (2023). Physical performance testing in climbing—a systematic review. Front Sports Act Living.

[49] Stien N, Saeterbakken AH, Andersen V (2022). Tests and procedures for measuring endurance, strength, and power in climbing-a mini-review. Front Sports Act Living.

[50] Jones EJ (2008). Cross-sectional area and muscular strength: a brief review. Sports Med.

[51] Draper N (2021). Performance assessment for rock climbers: the international rock climbing research association sport-specific test battery. Int J Sports Physiol Perform.

[52] Ruben RM (2010). The acute effects of an ascending squat protocol on performance during horizontal plyometric jumps. J Strength Cond Res.

[53] Seitz LB, de Villarreal ES, Haff GG (2014). The temporal profile of postactivation potentiation is related to strength level. J Strength Cond Res.

[54] Suchomel TJ (2018). The importance of muscular strength: training considerations. Sports Med.

[55] Stone MH (2002). How much strength is necessary?. Phys Ther Sport.

[56] Wisloff U (2004). Strong correlation of maximal squat strength with sprint performance and vertical jump height in elite soccer players. Br J Sports Med.

[57] Suchomel TJ (2016). Potentiation following ballistic and nonballistic complexes: the effect of strength level. J Strength Cond Res.

[58] Keiner M (2013). Strength performance in youth: trainability of adolescents and children in the back and front squats. J Strength Cond Res.

[59] Kraemer WJ, Newton RU (2000). Training for muscular power. Phys Med Rehabil Clin N Am.

[60] Stone MH (2022). Training specificity for athletes: emphasis on strength-power training: a narrative review. J Funct Morphol Kinesiol.

[61] Cormie P, McGuigan MR, Newton RU (2010). Adaptations in athletic performance after ballistic power versus strength training. Med Sci Sports Exerc.

[62] Cormie P, McGuigan MR, Newton RU (2010). Changes in the eccentric phase contribute to improved stretch-shorten cycle performance after training. Med Sci Sports Exerc.

[63] Cormie P, McBride JM, McCaulley GO (2009). Power-time, force-time, and velocity-time curve analysis of the countermovement jump: impact of training. J Strength Cond Res.

[64] Lake JP (2021). Effect of barbell load on vertical jump landing force-time characteristics. J Strength Cond Res.

[65] Dankel SJ (2017). Muscle adaptations following 21 consecutive days of strength test familiarization compared with traditional training. Muscle Nerve.

[66] Mattocks KT (2017). Practicing the test produces strength equivalent to higher volume training. Med Sci Sports Exerc.

[67] La Scala Teixeira CV (2017). "You're only as strong as your weakest link": a current opinion about the concepts and characteristics of functional training. Front Physiol.

[68] Saul D (2019). Determinants for success in climbing: a systematic review. J Exerc Sci Fit.

[69] Wackerhage H (2019). Stimuli and sensors that initiate skeletal muscle hypertrophy following resistance exercise. J Appl Physiol.

[70] Krieger JW (2010). Single vs. multiple sets of resistance exercise for muscle hypertrophy: a meta-analysis. J Strength Cond Res.

[71] Ralston GW (2017). The effect of weekly set volume on strength gain: a meta-analysis. Sports Med.

[72] American College of Sports, M., American college of sports medicine position stand. Progression models in resistance training for healthy adults. Med Sci Sports Exerc, 2009; **41**(3): 687–708. 10.1249/MSS.0b013e3181915670 19204579

[73] Androulakis-Korakakis P, Fisher JP, Steele J (2020). The minimum effective training dose required to increase 1RM strength in resistance-trained men: a systematic review and meta-analysis. Sports Med.

[74] Schoenfeld BJ (2015). Effects of low- vs. high-load resistance training on muscle strength and hypertrophy in well-trained men. J Strength Cond Res.

[75] Schoenfeld BJ (2013). Is there a minimum intensity threshold for resistance training-induced hypertrophic adaptations?. Sports Med.

[76] Jenkins NDM (2017). Greater neural adaptations following high- vs low-load resistance training. Front Physiol.

[77] Folland JP, Williams AG (2007). The adaptations to strength training: morphological and neurological contributions to increased strength. Sports Med.

[78] Del Vecchio A (2019). The increase in muscle force after 4 weeks of strength training is mediated by adaptations in motor unit recruitment and rate coding. J Physiol.

[79] Zourdos MC (2016). Efficacy of daily 1RM training in well-trained powerlifters and weightlifters: a case series. Nutr Hosp.

[80] Saeterbakken AH (2016). Resistance-training exercises with different stability requirements: time course of task specificity. Eur J Appl Physiol.

[81] Behm DG, Sale DG (1993). Intended rather than actual movement velocity determines velocity-specific training response. J Appl Physiol.

[82] Fyfe JJ, Hamilton DL, Daly RM (2022). Minimal-dose resistance training for improving muscle mass, strength, and function: a narrative review of current evidence and practical considerations. Sports Med.

[83] Barbalho M (2019). Evidence for an upper threshold for resistance training volume in trained women. Med Sci Sports Exerc.

[84] Peterson MD, Rhea MR, Alvar BA (2004). Maximizing strength development in athletes: a meta-analysis to determine the dose-response relationship. J Strength Cond Res.

[85] Behm DG (2023). Minimalist training: Is lower dosage or intensity resistance training effective to improve physical fitness? A narrative review. Sports Med.

[86] Fisher J, Steele J, Smith D (2017). High- and low-load resistance training: interpretation and practical application of current research findings. Sports Med.

[87] Choi J (1998). The difference between effects of power-up type and bulk-up type strength training exercises - with special reference to muscle cross-sectional area, muscular strength, anaerobic power and anaerobic endurance. Jpn J Phys Fitness Sports Med.

[88] Masuda K (1999). Maintenance of myoglobin concentration in human skeletal muscle after heavy resistance training. Eur J Appl Physiol Occup Physiol.

[89] Mitchell CJ (2012). Resistance exercise load does not determine training-mediated hypertrophic gains in young men. J Appl Physiol.

[90] Burd NA (2010). Low-load high volume resistance exercise stimulates muscle protein synthesis more than high-load low volume resistance exercise in young men. PLoS ONE.

[91] Ronnestad BR, Mujika I (2014). Optimizing strength training for running and cycling endurance performance: a review. Scand J Med Sci Sports.

[92] White DJ, Olsen PD (2010). A time motion analysis of bouldering style competitive rock climbing. J Strength Cond Res.

[93] Mermier CM (2000). Physiological and anthropometric determinants of sport climbing performance. Br J Sports Med.

[94] Fisher J, Steele J, Smith D (2013). Evidence-based resistance training recommendations for muscular hypertrophy. Med Sport.

[95] Schoenfeld BJ (2016). Muscular adaptations in low- versus high-load resistance training: a meta-analysis. Eur J Sport Sci.

[96] Pope ZK, Willardson JM, Schoenfeld BJ (2013). Exercise and blood flow restriction. J Strength Cond Res.

[97] Takarada Y, Takazawa H, Ishii N (2000). Applications of vascular occlusion diminish disuse atrophy of knee extensor muscles. Med Sci Sports Exerc.

[98] Javorsky T (2023). Comparing low volume of blood flow restricted to high-intensity resistance training of the finger flexors to maintain climbing-specific strength and endurance: a crossover study. Front Sports Act Living.

[99] Moran-Navarro R (2017). Time course of recovery following resistance training leading or not to failure. Eur J Appl Physiol.

[100] Buckner SL (2021). Do exercise-induced increases in muscle size contribute to strength in resistance-trained individuals?. Clin Physiol Funct Imaging.

[101] Androulakis-Korakakis P (2018). Reduced volume 'daily max' training compared to higher volume periodized training in powerlifters preparing for competition-a pilot study. Sports.

[102] Izquierdo M (2006). Differential effects of strength training leading to failure versus not to failure on hormonal responses, strength, and muscle power gains. J Appl Physiol.

[103] Ralston GW (2018). Weekly training frequency effects on strength gain: a meta-analysis. Sports Med Open.

[104] Drinkwater EJ (2005). Training leading to repetition failure enhances bench press strength gains in elite junior athletes. J Strength Cond Res.

[105] Refalo MC (2022). Towards an improved understanding of proximity-to-failure in resistance training and its influence on skeletal muscle hypertrophy, neuromuscular fatigue, muscle damage, and perceived discomfort: A scoping review. J Sports Sci.

[106] Hartman MJ (2007). Comparisons between twice-daily and once-daily training sessions in male weight lifters. Int J Sports Physiol Perform.

[107] Pedersen H (2022). Effects of one long vs. two short resistance training sessions on training volume and affective responses in resistance-trained women. Front Psychol.

[108] Correa DA (2022). Twice-daily sessions result in a greater muscle strength and a similar muscle hypertrophy compared to once-daily session in resistance-trained men. J Sports Med Phys Fitness.

[109] Hartman MJ (2007). Comparisons between twice-daily and once-daily training sessions in male weight lifters. Int J Sports Physiol Perform.

[110] Gonzalez-Badillo JJ, Izquierdo M, Gorostiaga EM (2006). Moderate volume of high relative training intensity produces greater strength gains compared with low and high volumes in competitive weightlifters. J Strength Cond Res.

[111] Cuthbert M (2021). Effects of variations in resistance training frequency on strength development in well-trained populations and implications for in-season athlete training: a systematic review and meta-analysis. Sports Med.

[112] Lundberg TR (2022). The effects of concurrent aerobic and strength training on muscle fiber hypertrophy: a systematic review and meta-analysis. Sports Med.

[113] Philippe M (2019). The effects of 8 weeks of two different training methods on on-sight lead climbing performance. J Sports Med Phys Fitness.

[114] Losnegard T (2011). The effect of heavy strength training on muscle mass and physical performance in elite cross country skiers. Scand J Med Sci Sports.

[115] Ronnestad BR, Hansen EA, Raastad T (2010). Effect of heavy strength training on thigh muscle cross-sectional area, performance determinants, and performance in well-trained cyclists. Eur J Appl Physiol.

[116] Hickson RC (1980). Interference of strength development by simultaneously training for strength and endurance. Eur J Appl Physiol Occup Physiol.

[117] Ozimek M (2016). Analysis of tests evaluating sport climbers' strength and isometric endurance. J Hum Kinet.

[118] Draper N (2011). Sport-specific power assessment for rock climbing. J Sports Med Phys Fitness.

[119] Vigouroux L (2019). Performing pull-ups with small climbing holds influences grip and biomechanical arm action. J Sports Sci.

[120] Laffaye G (2014). Upper-limb power test in rock-climbing. Int J Sports Med.

[121] Levernier G, Samozino P, Laffaye G (2020). Force–velocity–power profile in high-elite boulder, lead, and speed climber competitors. Int J Sports Physiol Perform.

[122] Stien N (2021). Upper body rate of force development and maximal strength discriminates performance levels in sport climbing. PLoS ONE.

[123] Schoenfeld BJ (2021). Loading recommendations for muscle strength, hypertrophy, and local endurance: a re-examination of the repetition continuum. Sports.

[124] Stien N (2021). The effects of prioritizing lead or boulder climbing among intermediate climbers. Front Sports Act Living.

[125] Sunde A (2019). Stronger is better: the impact of upper body strength in double poling performance. Front Physiol.

[126] Berryman N (2018). Strength training for middle- and long-distance performance: a meta-analysis. Int J Sports Physiol Perform.

[127] Aagaard P (2011). Effects of resistance training on endurance capacity and muscle fiber composition in young top-level cyclists. Scand J Med Sci Sports.

[128] Michailov M (2017). Physiological responses during two climbing tests with different hold types. Int J Sports Sci Coach.

[129] Schoffl V (2010). Evaluation of injury and fatality risk in rock and ice climbing. Sports Med.

[130] Lhee SH (2021). Sports injury and illness incidence among South Korean elite athletes in the 2018 Asian games: a single-physician prospective study of 782 athletes. BMJ Open Sport Exerc Med.

[131] Franco MF (2021). Prevalence of overuse injuries in athletes from individual and team sports: a systematic review with meta-analysis and GRADE recommendations. Braz J Phys Ther.

[132] Prieto-Gonzalez P (2021). Epidemiology of sports-related injuries and associated risk factors in adolescent athletes: an injury surveillance. Int J Environ Res Public Health.

[133] Gabbett TJ (2016). The training—injury prevention paradox: should athletes be training smarter and harder?. Br J Sports Med.

[134] Kennedy MD (2012). Can pre-season fitness measures predict time to injury in varsity athletes? A retrospective case control study. Sports Med Arthrosc Rehabil Ther Technol.

[135] Lauersen JB, Bertelsen DM, Andersen LB (2014). The effectiveness of exercise interventions to prevent sports injuries: a systematic review and meta-analysis of randomised controlled trials. Br J Sports Med.

[136] Woollings KY (2015). Incidence, mechanism and risk factors for injury in youth rock climbers. Br J Sports Med.

[137] Josephsen G (2007). Injuries in bouldering: a prospective study. Wilderness Environ Med.

[138] Lehnhard RA, Lehnhard HR, Young R (1996). Monitoring injuries on a college soccer team: the effect of strength training. J Strength Cond Res.

[139] Fleck SJ, Falkel JE (1986). Value of resistance training for the reduction of sports injuries. Sports Med.

[140] Joubert L (2022). Prevalence of amenorrhea in elite female competitive climbers. Front Sports Act Living.

[141] Gronhaug G, Norberg M (2016). First overview on chronic injuries in sport climbing: proposal for a change in reporting of injuries in climbing. BMJ Open Sport Exerc Med.

[142] Gronhaug G (2018). Self-reported chronic injuries in climbing: who gets injured when?. BMJ Open Sport Exerc Med.

[143] Gracey E (2020). Tendon and ligament mechanical loading in the pathogenesis of inflammatory arthritis. Nat Rev Rheumatol.

[144] Laursen PB (2010). Training for intense exercise performance: high-intensity or high-volume training?. Scand J Med Sci Sports.

[145] Grgic J (2018). Effect of resistance training frequency on gains in muscular strength: a systematic review and meta-analysis. Sports Med.

[146] Figueiredo VC, de Salles BF, Trajano GS (2018). Volume for muscle hypertrophy and health outcomes: the most effective variable in resistance training. Sports Med.

[147] Philippe AG (2015). Modeling the responses to resistance training in an animal experiment study. Biomed Res Int.

[148] Schoenfeld BJ, Ogborn D, Krieger JW (2017). The dose-response relationship between resistance training volume and muscle hypertrophy: are there really still any doubts?. J Sports Sci.

[149] Fisher JP, Steele J (2017). Heavier and lighter load resistance training to momentary failure produce similar increases in strength with differing degrees of discomfort. Muscle Nerve.

[150] Buckner SL (2018). The affective and behavioral responses to repeated "strength snacks". Physiol Int.

[151] Ozimek M (2017). Somatic profile of the elite boulderers in Poland. J Strength Cond Res.

[152] Gronhaug G (2019). Lean and mean? Associations of level of performance, chronic injuries and BMI in sport climbing. BMJ Open Sport Exerc Med.

